# Machine Learning–Based Explainable Automated Nonlinear Computation Scoring System for Health Score and an Application for Prediction of Perioperative Stroke: Retrospective Study

**DOI:** 10.2196/58021

**Published:** 2025-03-19

**Authors:** Mi-Young Oh, Hee-Soo Kim, Young Mi Jung, Hyung-Chul Lee, Seung-Bo Lee, Seung Mi Lee

**Affiliations:** 1 Department of Neurology Sejong General Hospital Sejong General Hospital Bucheon-si Republic of Korea; 2 Department of Medical Informatics School of Medicine Keimyung University Daegu Republic of Korea; 3 Department of Obstetrics and Gynecology Seoul National University Bundang Hospital Seongnam Republic of Korea; 4 Department of Anesthesiology and Pain Medicine Seoul National University College of Medicine Seoul Republic of Korea; 5 Department of Anesthesiology and Pain Medicine Seoul National University Hospital Seoul Republic of Korea; 6 Department of Obstetrics and Gynecology College of Medicine Seoul National University Seoul Republic of Korea; 7 Department of Obstetrics and Gynecology Seoul National University Hospital Seoul Republic of Korea; 8 Innovative Medical Technology Research Institute Seoul National University Hospital Seoul Republic of Korea; 9 Institute of Reproductive Medicine and Population & Medical Big Data Research Center Seoul National University Seoul Republic of Korea

**Keywords:** machine learning, explainability, score, computation scoring system, Nonlinear computation, application, perioperative stroke, perioperative, stroke, efficiency, ML-based models, patient, noncardiac surgery, noncardiac, surgery, effectiveness, risk tool, risk, tool, real-world data

## Abstract

**Background:**

Machine learning (ML) has the potential to enhance performance by capturing nonlinear interactions. However, ML-based models have some limitations in terms of interpretability.

**Objective:**

This study aimed to develop and validate a more comprehensible and efficient ML-based scoring system using SHapley Additive exPlanations (SHAP) values.

**Methods:**

We developed and validated the Explainable Automated nonlinear Computation scoring system for Health (EACH) framework score. We developed a CatBoost-based prediction model, identified key features, and automatically detected the top 5 steepest slope change points based on SHAP plots. Subsequently, we developed a scoring system (EACH) and normalized the score. Finally, the EACH score was used to predict perioperative stroke. We developed the EACH score using data from the Seoul National University Hospital cohort and validated it using data from the Boramae Medical Center, which was geographically and temporally different from the development set.

**Results:**

When applied for perioperative stroke prediction among 38,737 patients undergoing noncardiac surgery, the EACH score achieved an area under the curve (AUC) of 0.829 (95% CI 0.753-0.892). In the external validation, the EACH score demonstrated superior predictive performance with an AUC of 0.784 (95% CI 0.694-0.871) compared with a traditional score (AUC=0.528, 95% CI 0.457-0.619) and another ML-based scoring generator (AUC=0.564, 95% CI 0.516-0.612).

**Conclusions:**

The EACH score is a more precise, explainable ML-based risk tool, proven effective in real-world data. The EACH score outperformed traditional scoring system and other prediction models based on different ML techniques in predicting perioperative stroke.

## Introduction

The risk scoring systems have been proposed to prognosticate critical medical conditions, aiming to identify high-risk patients who are likely to experience adverse outcomes [1-6]. Traditionally, risk-scoring systems have been developed using conventional statistical approaches, based on the assumption of linearity between variables and outcomes [7,8]. This method has provided a comprehensive understanding of patient risk profiles, but it may not fully capture complex, nonlinear interactions, potentially leading to less accurate risk assessments. Furthermore, the clinical variables incorporated into these systems were typically ascertained through univariate analysis, enriched by insights from expert opinions, or selected from a range of risk factors established in previous literature [8]. Consequently, these systems faced limitations in rapidly integrating state-of-the-art medical knowledge alongside medical advancements [9-11]. With the rapid expansion and increasing diversity of medical data, these limitations have become more pronounced [10].

To address these limitations, machine learning (ML) techniques have emerged as promising avenues for creating new and diverse risk models by leveraging extensive electronic medical records [12,13]. Despite their exceptional predictive performance, the lack of interpretability has limited their adoption in real-world medical practice [14]. Recent efforts in ML have addressed the “black box” issue of existing models by presenting data in a more understandable fashion [15]. For instance, an ML-based automated scoring model was developed by integrating the Random Forest (RF) algorithm and logistic regression methods [16]. This model was designed to be easy to understand and apply to various clinical situations, such as in-hospital mortality and out-of-hospital cardiac arrest [16,17]. However, these approaches still rely on scoring methods rooted in traditional statistics, using variables selected by the ML models [16]. Furthermore, the generalizability and scalability of ML models are hampered by limited external validation. Therefore, there is a compelling need to develop and validate a fully automated ML-based scoring system that can address nonlinearity assumptions.

In response to these challenges, this research aimed to develop and validate an Explainable Automated nonlinear Computation scoring system for Health (EACH) score. This system addresses nonlinearity assumptions and enhances explainability. In addition, we applied the EACH score to predict perioperative stroke to assess its performance in real-world clinical practice and examined its performance compared with traditional scores and other ML-based scoring systems.

Perioperative stroke significantly impacts postoperative morbidity and mortality. Patients who experience perioperative stroke are more likely to develop postoperative delirium, have prolonged hospitalization, increased in-hospital mortality, and be discharged to nursing facilities [18-20]. However, the detection of perioperative stroke is challenging due to the effects of anesthetic and analgesic medications, which contribute to a low rate of hyperacute thrombolytic treatment [21]. Therefore, to reduce the risk of perioperative stroke, it is essential to use more effective tools for identifying high-risk patients and to implement close monitoring of these patients. To apply the EACH score in predicting perioperative stroke, we aimed to develop and validate a more accurate and explainable prediction model that can be easily adapted to real-world practice.

## Methods

### Overview

**Figure 1:**
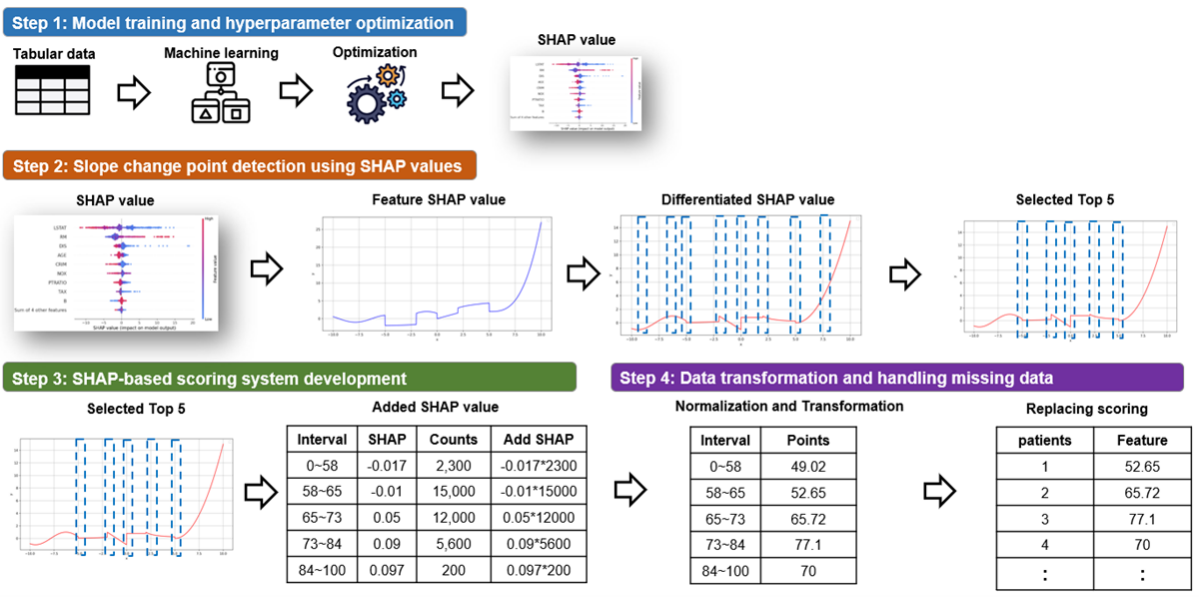
Visual guide to the sequential steps and their detailed execution. SHAP: SHapley Additive exPlanations.

We developed and validated the EACH, designed to automate the development of clinical scoring models for predefined health outcomes. The development and application of EACH were segmented into several steps, as illustrated in Figure 1. We tried to follow the guideline for developing reporting machine learning prediction models in biomedical research [22].

### Step 1: Model Training and Hyperparameter Optimization

When implementing the EACH score, the initial step involved training the CatBoost model with hyperparameter optimization for each set of clinical data, followed by determination of important features using SHapley Additive exPlanations (SHAP) values [23].

### Step 2: The Detection of Slope Change Point in Plots of SHAP Values and Selection of the Top 5 Steepest Slope Change Points

In this step, we harnessed the power of automation to identify the critical features that influence the accuracy of the prediction model. The process began with the generation of SHAP value plots for each feature, which visually represented the impact of these features on the model predictions. The key advantage of our approach is the automated detection of the slope change points within these plots. The slope change points are significant shifts in the importance of each variable, indicating where the influence of the variable undergoes a notable change.

**Figure 2:**
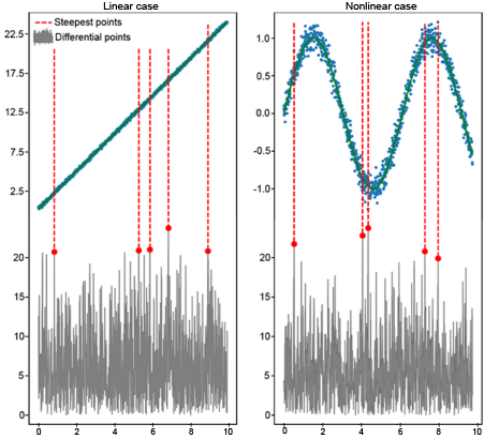
Differential slope change comparison: linear versus nonlinear cases.

The algorithm identified the 5 most pronounced slope change points for each feature. These critical points indicate where the model’s sensitivity to feature values is dramatically altered, thus playing a pivotal role in subsequent feature scoring. Figure 2 illustrates this method by contrasting linear and nonlinear cases, where the latter demonstrates the effectiveness of the method in recognizing complex nonlinear relationships often present in clinical data.

By isolating these key intervals, we delineated the feature value ranges that were most influential on the model’s predictions, thus allowing for a more accurate SHAP-based scoring system.

### Step 3: SHAP-Based Scoring System Development

The third step shows how to generate scores using the SHAP values for each variable. The score was calculated by adding the SHAP values of the data points obtained from each section to accurately quantify the impact of individual intervals for each feature. This was for the model’s sensitive interpretation of the different ranges within the variable and was reflected in the final scoring system.

### Step 4: Data Transformation and Handling Missing Data

#### Normalization of Scores Across Features

In this step, we focused on standardizing the scoring system across all features. This normalization process ensured that the scores from different features were comparable and appropriately weighted within the overall scoring model. Normalization is based on the total range of scores across all features, thereby aligning them on a unified scale.

#### Handling Missing Data With Interval Averaging

If the actual values for a particular interval were unavailable, our model adopted a fallback strategy that uses the average of values in adjacent intervals. If there is no data in the interval immediately next to the missing value, the entire range of the interval was used to preserve the integrity of the model’s risk assessment in the face of data sparsity.

### Clinical Study Design

In applying the EACH score to assess stroke risk during the perioperative period in noncardiac surgeries, we approached our clinical study design with a focus on real-world applicability. This study was structured using 3 datasets: training, internal validation, and external validation. The training and internal validation datasets were derived from patient records at Seoul National University Hospital (SNUH) from 2016 to 2019. For a broader perspective and to assess the performance of the model in different settings, we included a geotemporal external validation using data from surgeries performed at the Boramae Medical Center (BMC) between 2020 and 2021.

A key aspect of our data preparation involved addressing missing values in preoperative variables. Recognizing the potential impact of incomplete data on the model’s accuracy, we used a methodological imputation approach. For continuous variables, missing values were imputed using their mean, whereas categorical variables were imputed using the mode [24].

**Figure 3:**
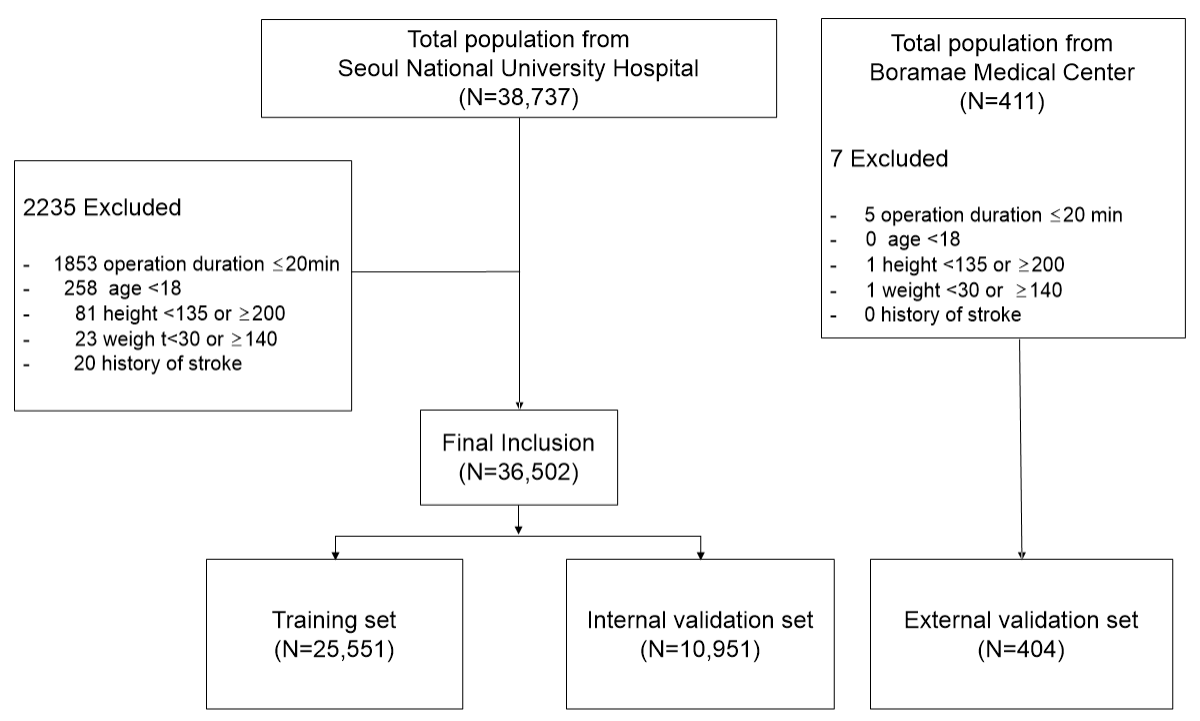
Flowchart of study population.

Patients were excluded from the study if the surgery lasted less than 20 minutes, if they were younger than 18 years or weighed less than 30 kg or more than 140 kg, if their height was outside the 135 cm to 200 cm range, or if they had a previous history of stroke. The developmental set consisted of 36,502 patients from Seoul National University Hospital. After applying the exclusion criteria, 404 patients from Boramae Medical Center were included in the external validation set (Figure 3). Perioperative stroke was defined as an ischemic brain infarction occurring within 30 days postoperatively and was identified through new ischemic lesions on diffusion-weighted imaging [18,21]. An experienced neurologist [M-YO] confirmed the stroke diagnosis by reviewing the imaging outcomes.

### Data Collection

#### Demographics and Comorbidities

We recorded patients' age, sex, physical metrics (height, weight, and body mass index [BMI]), and a range of preexisting conditions, including hypertension, diabetes (with or without insulin medication), previous cardiovascular events, asthma, chronic obstructive pulmonary disease, liver and kidney diseases, and tuberculosis.

#### Surgical Information

Surgical risk was classified according to the American Society of Anesthesiologists (ASA) standards. Information on whether the surgery was emergent and the type was collected.

#### Preoperative Laboratory Findings

A comprehensive set of laboratory results were collected, including hemoglobin levels, renal function indicators (blood urea nitrogen, creatinine, and estimated glomerular filtration rate), nutritional markers (albumin), electrolytes (sodium and potassium), glucose levels, liver enzymes (aspartate aminotransferase and alanine aminotransferase), platelet count, and coagulation status assessed by partial thromboplastin time.

#### Revised Cardiac Risk Index

This traditional scoring system is used to assess the risk of major adverse cardiac events including stroke in noncardiac surgery. The Revised Cardiac Risk Index (RCRI) is composed of the factors mentioned above, such as type of surgery, history of ischemic heart disease, congestive heart failure, cerebrovascular disease, preoperative treatment with insulin, and a preoperative creatinine level greater than 2 mg/dL [13]. The RCRI was used as the comparative score.

### Prediction Model Based on Other ML Techniques

We developed prediction model based on several ML algorithms, including Support Vector Machine (SVM) [25], Decision Tree Classifier (DTC) [26], RF [27], and CatBoost [28] to compare the performance with the EACH score. The developmental dataset was systematically divided into a training set comprising 70% of the data for model development, and the remaining 30% formed the test set used to assess model performance. Hyperparameter optimization for each model was performed using grid-search cross-validation to identify the optimal settings that maximize the discriminative power of the model [29].

### External Validation

External validation was conducted using the BMC cohort to assess the prediction performance of the EACH score, which was developed to predict perioperative stroke based on the SNUH cohort. The data used for validation were entirely separate from the data used during the development of the EACH score. BMC cohort was geographically, and temporally different cohort from the development set, SNUH cohort.

### Statistical Analysis

Continuous variables were analyzed using the Student *t* test and the Mann-Whitney *U* test to determine the significance of differences between the 2 groups.

Categorical variables in both datasets were evaluated using the chi-square test to investigate the presence of significant associations or discrepancies between the categories. All tests were 2-sided, and statistical significance was set at *P*<.05. ML modeling was performed in python 3.8 using the Scikit-Learn package [30]. The area under the receiver operating characteristic curve (AUC), accuracy, sensitivity, positive predictive value (PPV), and negative predictive value (NPV) were calculated to evaluate the performance of the prediction model [31].

### Ethical Considerations

This study was approved by the institutional review board (IRB) of Seoul National University Hospital on April 7, 2020 (IRB Number, 2003-067-1108), and Boramae Medical Center on January 7, 2021 (IRB Number, 30-2021-4). The IRB determined that participant consent was waived in this retrospective study. We tried to follow the tripod guideline [32].

## Results

### Baseline Characteristics of the Study Cohorts

Our retrospective observational cohort study used data from SNUH comprising the training set (25,551 cases) and the internal validation set (10,951 cases) and the data of 404 patients from BMC to externally validate the prediction model in a geographically and temporally different population. The baseline characteristics of patients in the SNUH and BMC cohorts are summarized in Table 1. Patients in the SNUH cohort were younger than those in the BMC cohort. The prevalence of diabetes was slightly higher in the BMC cohort. Hemoglobin, creatinine, and albumin levels were higher in patients in the SNUH cohort, whereas glucose levels were slightly higher in patients in the BMC cohort.

**Table 1:** Baseline characteristics.

Characteristics			SNUH^a^ cohort		BMC^b^ cohort		*P* value	
Number of strokes, n (%)			139 (0.38)		10 (2.48)		<.001	
Age (years), mean (SD)			56.91 (15.23)		62.44 (14.57)		<.001	
Sex, male, n (%)			16,265 (55.44)		204 (50.50)		.48	
Height (cm), mean (SD)			161.97 (8.7)		160.7 (8.61)		.01	
Weight (kg), mean (SD)			63.46 (12.01)		63.36 (11.91)		.10	
BMI (kg/m^2^), mean (SD)			24.12 (3.73)		24.49 (3.93)		.04	
**Preoperative ASA^c^, n (%)**								.71
	1	10,778 (29.53)		25 (6.19)		—^d^		
	2	21,283 (58.31)		298 (73.76)		—		
	3	4172 (11.43)		74 (18.32)		—		
	4	252 (0.69)		7 (1.73)		—		
	5	17 (0.05)		0 (0)		—		
Emergency surgery, n (%)			2132 (5.84)		19 (4.7)		.53	
Preoperative hypertension, n (%)			11,395 (31.22)		168 (41.58)		.06	
Preoperative diabetes, n (%)			5543 (15.19)		92 (22.77)		.90	
Preoperative cardiovascular accident, n (%)			729 (2.00)		26 (6.44)		>.99	
Preoperative asthma, n (%)			230 (0.63)		8 (1.98)		>.99	
Preoperative COPD^e^, n (%)			234 (0.64)		7 (1.73)		.15	
Preoperative liver disease, n (%)			1437 (3.94)		42 (10.40)		.69	
Preoperative kidney disease, n (%)			1179 (3.23)		39 (9.65)		.97	
Preoperative tuberculosis, n (%)			366 (1.00)		26 (6.44)		.91	
Surgery type^f^, n (%)			19,787 (54.21)		212 (52.48)		.51	
Hemoglobin (g/dL), mean (SD)			12.90 (1.86)		12.08 (1.93)		<.001	
Platelet (×10^3^/µL), mean (SD)			245.18 (79.48)		247.54 (87.81)		.86	
Blood urea nitrogen (mg/dL), mean (SD)			15.82 (9.18)		16.88 (11.59)		.34	
Creatinine (mg/dL), mean (SD)			1.01 (1.34)		0.43 (1.55)		<.001	
Albumin (g/dL), mean (SD)			4.05 (0.47)		3.75 (0.6)		<.001	
Sodium (mmol/L), mean (SD)			140.17 (2.66)		138.85 (3.07)		<.001	
Potassium (mmol/L), mean (SD)			4.24 (0.41)		3.81 (0.54)		<.001	
Glucose (mg/dL), mean (SD)			114.88 (39.92)		121.52 (45.85)		.01	
Prothrombin time, n (%)			103.58 (14.86)		11.96 (1.12)		<.001	
Partial thromboplastin time (sec), mean (SD)			31.45 (5.56)		27.66 (3.9)		<.001	
Aspartate aminotransferase (IU/L), mean (SD)			25.33 (69.93)		25.32 (52.62)		.97	
Alanine aminotransferase (IU/L), mean (SD)			25.44 (52.62)		28.68 (32.74)		.01	
Estimated glomerular filtration rate (mL/min/1.73 m²), mean (SD)			83.79 (27.25)		83.83 (24.82)		.84	

^a^SNUH: Seoul National University Hospital.

^b^BMC: Boramae Medical Center.

^c^ASA: American Society of Anesthesiologists classification.

^d^Not applicable.

^e^COPD: chronic obstructive pulmonary disease.

^f^Surgery types included intrathoracic, intra-abdominal, and supra-inguinal vascular surgery.

### Scoring System Based on Clinical Feature Intervals

The entire scoring system is presented in Multimedia Appendix 1. Scores ranging from 0 to 100 were assigned to the clinical features based on specific intervals determined by the slope change points. For example, albumin levels less than 4.1 g/dL were associated with a higher risk of perioperative stroke. Specifically, the highest score of 55.4 was assigned to the 2.4-3.5 g/dL range, indicating a particularly elevated risk for some low albumin levels. Similarly, hemoglobin levels were segmented, with the highest score of 51.5 assigned to the 5.0-11.0 g/dL range. In addition, the 18.6-20.7 kg/m² interval of BMI received a score of 51.8, suggesting a higher risk associated with specific BMI levels. However, age was divided into intervals, with scores increasing incrementally from 49.0 for the 18-49 years age group to 51.8 for those aged 64-97 years old. Creatinine levels did not differ significantly between intervals. These results suggest that continuous variables could impact predictive performance in various nonlinear patterns and that this pattern is well reflected in the EACH scoring system. Categorical variables were scored on the basis of their relative importance in predicting perioperative stroke.

### Comparative Analysis of the Performance of EACH Scores Relative to Other ML Models

The EACH score demonstrated superior performance compared with other ML-based models, such as RF and CatBoost, as detailed in Table 2. The EACH score had the highest AUC (0.829) and sensitivity (0.881). The SVM, DTC, RF, and CatBoost models exhibited competitive yet slightly lower AUCs of 0.500, 0.501, 0.802, and 0.822, respectively. In the external validation, the EACH model consistently maintained high performance. It achieved an AUC of 0.784 and attained the highest sensitivity at 0.900 (Table 2).

**Table 2:** Comparison between the performance of Explainable Automated nonlinear Computation scoring system for Health (EACH) score and that of other machine learning models.

Models		AUC^a^	Accuracy	Sensitivity	Specificity	PPV^b^	NPV^c^
**Internal validation**							
	Support Vector Machine	0.500	0.954	0.888	0.268	0.003	0.999
	Decision Tree Classifier	0.501	0.961	0.820	0.566	0.007	0.999
	Random Forest	0.802	0.640	0.856	0.639	0.009	0.999
	CatBoost	0.822	0.686	0.770	0.686	0.010	0.999
	EACH^d^ score	0.829	0.655	0.881	0.654	0.010	0.999
**External validation**							
	Support Vector Machine	0.500	0.955	0.700	0.655	0.049	0.989
	Decision Tree Classifier	0.503	0.964	0.821	0.621	0.005	0.999
	Random Forest	0.744	0.797	0.600	0.802	0.071	0.988
	CatBoost	0.782	0.693	0.900	0.688	0.068	0.996
	EACH score	0.784	0.688	0.900	0.683	0.067	0.996

^a^AUC: area under the receiver operating characteristic curve.

^b^PPV: positive predictive value.

^c^NPV: negative predictive value.

^d^EACH score: Explainable Automated nonlinear Computation scoring system for Health.

### Comparative Analysis of the Performance of the EACH Explainable Score Relative to Traditional Scores

**Figure 4:**
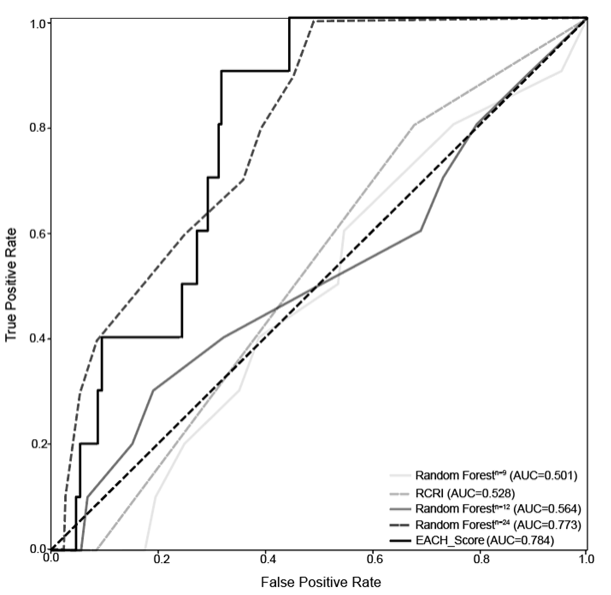
Comparison of receiver operating characteristic curves.

We compared the effectiveness of the EACH score with that of a traditional scoring system based on classical statistical methods and ML algorithms (Figure 4). The EACH score demonstrated superior performance compared with the RCRI, a scoring system used to predict perioperative stroke based on classical logistic regression analysis (AUC=0.528 vs AUC=0.784).

### Comparative Analysis of the Performance of the EACH Score Relative to Other ML-Based Scoring Systems

In addition, we compared the performance of the EACH score with that of the ML-based score generation system, AutoScore (eg, RF^n=12, n=24^). The performance of AutoScore increased with the number of features: with 9 features, the AUC was 0.501; with 12 features, the AUC was 0.564; and with 24 features, the AUC was 0.773. However, the EACH score surpassed the performance of AutoScore, demonstrating a superior AUC of 0.784 (Figure 4).

### Risk Stratification Using the EACH Score: Low- Versus High-Risk Patients for Perioperative Stroke

The 2 patients representing low and high perioperative stroke risk based on the EACH score are shown in Table 3. The table illustrates how the EACH score model assigned risk scores to individual patient characteristics, leading to a cumulative risk assessment. In assigning scores to patients, the low-risk case, characterized by younger age (42.77 years) and moderate BMI (24.02 kg/m^2^), tended to have lower ASA scores, no emergency operation, and no premorbidities. The laboratory results included slightly higher hemoglobin levels (13 g/dL) and higher platelet counts (293 ×10^3^/µL), resulting in a lower total score (1379.16) indicative of a lower risk profile. Conversely, the high-risk patient was characterized by older age (71.07 years), lower BMI (22.84 kg/m^2^), higher ASA scores, emergency operation, and surgery with a high risk for cardiovascular complications. The patient had lower hemoglobin levels (12 g/dL), lower platelet counts (92 ×10^3^/µL), lower albumin levels (3 g/dL), and higher glucose (115 mg/dL) levels. These factors contributed to the higher total score (1517.29) of this patient, indicating a higher-risk profile. The key distinguishing factors in risk assessment included age, ASA score, surgery type, and laboratory values. The model integrated a wide range of clinical variables into a single risk assessment metric. The ability of the EACH score to distill complex clinical data into a quantifiable stroke risk assessment provides a clear and actionable tool for perioperative stroke risk stratification.

**Table 3:** Comparative risk stratification using Explainable Automated nonlinear Computation scoring system for Health score: low versus high-risk patients for perioperative stroke.

Characteristic	Actual value	Low risk score	Actual value	High risk score
Age	42.77	49.02	71.07	51.82
Sex, male	1	48.52	1	48.52
Height	160.4	49.78	156.3	49.91
Weight	61.8	48.60	55.8	53.02
BMI	24.02	48.85	22.84	48.85
Preoperative ASA^a^	1	46.17	4	53.76
Emergency operation	0	47.10	1	52.95
Preoperative hypertension	0	49.30	0	49.30
Preoperative diabetes	0	49.83	0	49.83
Preoperative cardiovascular accident	0	47.39	0	47.39
Preoperative asthma	0	50.00	0	50.00
Preoperative COPD^b^	0	49.59	0	49.59
Preoperative liver disease	0	49.81	1	50.17
Preoperative kidney disease	0	49.80	0	49.80
Preoperative tuberculosis	0	49.80	0	49.80
Surgery type^c^	0	0	1	100
Hemoglobin	13	49.66	12	49.49
Platelet	293	49.80	92	50.66
Blood urea nitrogen	14	49.27	8	49.27
Creatinine	0	50.40	0	50.40
Albumin	4	50.54	3	55.35
Sodium	137	50.41	137	50.41
Potassium	4	53.46	3	50.75
Glucose	81	47.39	115	54.08
Prothrombin time	11.9	52.13	11.6	52.13
Partial thromboplastin time	28	46.54	28	46.54
Aspartate aminotransferase	14	47.30	64	52.37
Alanine aminotransferase	22	49.64	78	50.30
Estimated glomerular filtration rate	120	49.06	90	50.83
Total scoring	—^d^	1379.16	—^d^	1517.29

^a^ASA: American Society of Anesthesiologists.

^b^COPD: chronic obstructive pulmonary disease.

^c^Surgery type included several type of surgeries Intrathoracic, intraabdominal, and supra-inguinal vascular surgery.

^d^Not applicable.

## Discussion

### Principal Findings

In this study, we developed and validated a comprehensible automated scoring system, the EACH score, using CatBoost. This system, when applied to predict perioperative stroke, showed superior performance compared with other ML models. It also outperformed the traditional risk score, the RCRI score, and other ML-based risk-scoring systems, such as AutoScore. Furthermore, the EACH score effectively discriminated between patients with high- and low-risk perioperative stroke.

Recent advancements in ML-based models have shown promising performance by integrating diverse datasets, irrespective of their linearity or nonlinearity. These models have the potential to overcome the limitations inherent in traditional statistical methods. However, broader application of these models is often restricted because of their lack of explainability and insufficient external validation [33,34].

### Comparison With Previous Work

To address these issues, Xie et al [16] developed AutoScore, a scoring generator that combines RF-based approaches with traditional logistic regression. However, AutoScore may not fully capture complex clinical relationships or accurately represent biological contexts owing to methodological limitations and reliance on logistic regression [16,17].

The uniqueness of the present study lies in the development of an entirely ML-based scoring system. The EACH score is adaptable to a variety of data types without relying on predefined assumptions. In this system, the range of each continuous variable was determined by the inflection point on the plot using SHAP values. SHAP values demonstrate the key features and the absolute and relative predictive values of each feature within the model and are widely used to enhance the explainability of ML models [14,23]. The inflection point on the plot of SHAP values could be assumed to be a significant change of its predictive impact on outcome [35]. Using the above characteristics of SHAP values, we revealed that the importance of each feature did not increase linearly with its value, but rather showed various relationships. Consequently, the EACH score uniquely reflects the varying significance of identical features, thereby influencing the performance of the prediction model in a distinct manner. Another strength of the EACH score is its robust handling of missing data. If the data for an interval were missing, they were replaced with the average of the surrounding intervals’ values, maintaining score completeness and accuracy [24,36].

When applied to the real-world clinical data, EACH outperformed the traditional score and other ML-based systems. This demonstrates that this approach could contribute to the construction of a novel risk assessment tool that achieves high predictive accuracy and is intuitive for clinicians to interpret and apply in real-world practice.

Using the EACH score, clinicians can better differentiate between high-risk and low-risk patients. This allows for the allocation of limited medical resources to high-risk groups, enabling closer monitoring of perioperative stroke occurrence and improving prognosis through timely treatment.

Furthermore, the EACH score can indirectly provide optimal targets for minimizing perioperative stroke risk for key correctable laboratory indicators such as albumin, electrolyte, and glucose by highlighting detailed trends in blood tests that increase risk nonlinearly. For instance, the EACH score identifies an albumin level of 4.1 to 4.4 mg/dL as being associated with the lowest perioperative stroke risk. Adjusting albumin levels to this range could potentially reduce the incidence of perioperative stroke.

To date, there has not been a prediction model that provides treatment strategies for optimal thresholds of preoperative laboratory indicators. The EACH score would be helpful for clinicians in decision-making to reduce perioperative stroke in complex preoperative environments by providing the transparent and straightforward explanation of how it works. Nevertheless, it is crucial to interpret these findings cautiously within the context of each patient's individual situation and clinical context.

### Limitations

In addition, we addressed the second common limitation in the application of ML-based prediction models by validating the EACH score in external cohorts from a secondary medical center with different geographical locations, patient volumes, and characteristics from the developmental cohorts. The superior performance of the EACH score in the external validation cohort added robustness to its reliability. Despite these advancements, it is important to acknowledge that the efficacy of the EACH score is contingent on the quality and comprehensiveness of the input data. Incomplete or biased data can result in skewed results. However, the EACH score showed excellent performance even with imbalanced data, such as perioperative stroke, which is a rare disease [19,21]. Nevertheless, there is a need for ongoing updates and validation of the model across diverse clinical settings. Furthermore, because the current dataset comprises only Asian patients, it is imperative to prospectively validate the EACH score in multiracial cohorts to ensure its generalizability.

### Conclusion

The EACH score represents a substantial advancement in the development of efficient, explainable, and automated ML-based risk assessment tools. The universal format of the EACH score is easily applicable to various medical scenarios, thereby reducing the need for labor-intensive data collection. Once integrated with electronic medical records, it generates specific predictions of different adverse outcomes [11,37]. Future developments should aim to enhance its user-friendliness for clinicians, potentially through the creation of intuitive interfaces or decision support tools that simplify the interpretation of results [38-40].

## Data Availability

The data that support the findings of this study are available from the corresponding author upon reasonable request. In addition, the corresponding code for model development is accessible on GitHub in the repository.

## Appendix

Multimedia Appendix 1 Supplemental Table. Complete SHapley Additive exPlanations value scoring by feature interval.

## Abbreviations

ASA: American Society of Anesthesiologists
AUC: area under the receiver operating characteristic curve
BMC: Boramae Medical Center
EACH: Explainable Automated Non-linear Computation scoring system for Health
IRB: institutional review board
NPV: negative predictive value:
ML: machine learning
PPV: positive predictive value
RCRI: Revised Cardiac Risk Index
RF: Random Forest
SHAP: SHapley Additive exPlanations
SNUH: Seoul National University Hospital
SVM: Support Vector Machine
