# Supplementary material for: Machine Learning–Based Explainable Automated Nonlinear Computation Scoring System for Health Score and an Application for Prediction of Perioperative Stroke: Retrospective Study
Source: J Med Internet Res. 2025 Mar 19;27:e58021. doi: 10.2196/58021 (PMC11966079; doi:10.2196/58021)
Supplement: Multimedia Appendix 1 [file jmir_v27i1e58021_app1.docx]

|  | | | | Variables and interval | Score based on SHAP Value | | | | |  |  |
| --- | --- | --- | --- | --- | --- | --- | --- | --- | --- | --- | --- |
| **Age (years)** | | | | | | | | | |  |  |
|  | | 18 ~ 49 | | | 49.0 | | | | |  |  |
|  | | 49 ~ 55 | | | 49.9 | | | | |  |  |
|  | | 55 ~ 64 | | | 49.4 | | | | |  |  |
|  | | 64 ~ 65 | | | 49.9 | | | | |  |  |
|  | | 64 ~ 97 | | | 51.8 | | | | |  |  |
| **Height (cm)** | | | | | | | | | |  |  |
|  | 135.0 ~ 150.4 | | | | 50.3 | | | | |  |  |
|  | 150.4 ~ 151.6 | | | | 50.0 | | | | |  |  |
|  | 151.6 ~ 158.1 | | | | 49.9 | | | | |  |  |
|  | 158.1 ~ 160.6 | | | | 49.8 | | | | |  |  |
|  | 160.6 ~ 194.3 | | | | 50.1 | | | | |  |  |
| **Weight (kg)** | | | | | | | | | |  |  |
|  | 30.0 ~ 56.6 | | | | 53.0 | | | | |  |  |
|  | 56.6 ~ 65.2 | | | | 48.6 | | | | |  |  |
|  | 65.2 ~ 84.2 | | | | 49.0 | | | | |  |  |
|  | 84.2 ~ 135.0 | | | | 50.2 | | | | |  |  |
| **Estimated glomerular filtration rate**  **(mL/min/1.73m²)** | | | | | | | | | |  |  |
|  | | 0.4 ~ 61.3 | | | 50.5 | | | | |  |  |
|  | | 61.3 ~ 72.5 | | | 50.4 | | | | |  |  |
|  | | 72.5 ~ 85.3 | | | 50.7 | | | | |  |  |
|  | | 85.3 ~ 96.4 | | | 50.8 | | | | |  |  |
|  | | 96.4 ~ 109.0 | | | 48.7 | | | | |  |  |
|  | | 109.0 ~ 419.8 | | | 49.1 | | | | |  |  |
| **BMI^a^ (kg/m^2^)** | | | | | | | | | |  |  |
|  | | | | 11.7 ~ 18.6 | 51.1 | | | | |  |  |
|  | | | | 18.6 ~ 20.7 | 51.8 | | | | |  |  |
|  | | | | 20.7 ~ 21.2 | 50.5 | | | | |  |  |
|  | | | | 21.2 ~ 26.6 | 48.9 | | | | |  |  |
|  | | | | 26.6 ~ 27.5 | 49.4 | | | | |  |  |
|  | | | | 27.5 ~ 49.7 | 48.5 | | | | |  |  |
| **Hemoglobin (g/dL)** | | | | | | | | | |  |  |
|  | | | 5.0 ~ 11.0 | | 51.5 | | | | |  |  |
|  | | | 11.0 ~ 11.8 | | 49.7 | | | | |  |  |
|  | | | 11.8 ~ 12.9 | | 49.5 | | | | |  |  |
|  | | | 12.9 ~ 14.0 | | 49.7 | | | | |  |  |
|  | | | 14.0 ~ 20.4 | | 49.8 | | | | |  |  |
| **Platelet (x10^3^/µl)** | | | | | | | | | |  |  |
|  | 8.0 ~ 163.0 | | | | 50.7 | | | | |  |  |
|  | 163.0 ~ 165.0 | | | | 50.0 | | | | |  |  |
|  | 165.0 ~ 179.0 | | | | 49.9 | | | | |  |  |
|  | 179.0 ~ 233.0 | | | | | 49.7 | | | | |  |
|  | 233.0 ~ 245.0 | | | | 50.0 | | | | |  |  |
|  | 245.0 ~ 1263.0 | | | | 49.8 | | | | |  |  |
| **Albumin (g/dL)** | | | | | | | | | |  |  |
|  | 0.4 ~ 2.4 | | | | 50.5 | | | | |  |  |
|  | 2.4 ~ 3.5 | | | | 55.4 | | | | |  |  |
|  | 3.5 ~ 4.1 | | | | 50.5 | | | | |  |  |
|  | 4.1 ~ 4.4 | | | | 46.8 | | | | |  |  |
|  | 4.4 ~ 4.9 | | | | 47.2 | | | | |  |  |
|  | 4.9 ~ 5.7 | | | | 49.9 | | | | |  |  |
| **Blood urea nitrogen (mg/dL)** | | | | | | | | | |  |  |
|  | | | 2.0 ~ 14.0 | | 49.3 | | | | |  |  |
|  | | | 14.0 ~ 17.0 | | 49.5 | | | | |  |  |
|  | | | 17.0 ~ 20.0 | | 49.8 | | | | |  |  |
|  | | | 20.0 ~ 127.0 | | 51.1 | | | | |  |  |
| **Creatinine (mg/dL)** | | | | | | | | | |  |  |
|  | | | 0.15 ~ 0.59 | | 50.4 | | | | |  |  |
|  | | | 0.59 ~ 0.91 | | 49.5 | | | | |  |  |
|  | | | 0.91 ~ 7.33 | | 50.0 | | | | |  |  |
|  | | | 7.33 ~ 25.62 | | 50.4 | | | | |  |  |
| **Sodium (mmol/L)** | | | | | | | | | |  |  |
|  | | | 111.0 ~ 139.0 | | 50.4 | | | | |  |  |
|  | | | 139.0 ~ 142.0 | | 49.7 | | | | |  |  |
|  | | | 142.0 ~ 144.0 | | 49.9 | | | | |  |  |
|  | | | 144.0 ~ 160.0 | | 50.4 | | | | |  |  |
| **Potassium (mmol/L)** | | | | | | | | | |  |  |
|  | 2.3 ~ 3.5 | | | | 50.8 | | | | |  |  |
|  | 3.5 ~ 4.0 | | | | 53.5 | | | | |  |  |
|  | 4.0 ~ 4.1 | | | | 50.0 | | | | |  |  |
|  | 4.1 ~ 4.2 | | | | 49.7 | | | | |  |  |
|  | 4.2 ~ 4.5 | | | | 48.6 | | | | |  |  |
|  | 4.5 ~ 8.4 | | | | 47.5 | | | | |  |  |
| **Prothrombin time (%)** | | | | | | | | | |  |  |
|  | | | | 14.0 ~ 97.0 | 52.1 | | | | |  |  |
|  | | | | 97.0 ~ 100.0 | 50.0 | | | | |  |  |
|  | | | | 100.0 ~ 106.0 | 49.3 | | | | |  |  |
|  | | | | 106.0 ~ 118.0 | 48.9 | | | | |  |  |
|  | | | | 118.0 ~ 172.0 | 49.8 | | | | |  |  |
| **Partial thromboplastin time (sec)** | | | | | | | | | |  |  |
|  | 18.3 ~ 27.0 | | | | 61.9 | | | | |  |  |
|  | 27.0 ~ 31.3 | | | | 46.5 | | | | |  |  |
|  | 31.3 ~ 32.1 | | | | 47.8 | | | | |  |  |
|  | | 32.1 ~ 34.3 | | | 46.5 | | | | |  |  |
|  | | 34.3 ~ 34.6 | | | 49.7 | | | | |  |  |
|  | | 34.6 ~ 400.0 | | | 47.6 | | | | |  |  |
| **Glucose (mg/dL)** | | | | | | | | | |  |  |
|  | | | 1.0 ~ 77.0 | | 49.9 | | | | |  |  |
|  | | | 77.0 ~ 95.0 | | 47.4 | | | | |  |  |
|  | | | 95.0 ~ 97.0 | | 49.5 | | | | |  |  |
|  | | | 97.0 ~ 110.0 | | 49.1 | | | | |  |  |
|  | | | 110.0 ~ 114.0 | | 50.1 | | | | |  |  |
|  | | | 114.0 ~ 755.0 | | 54.1 | | | | |  |  |
| **Alanine aminotransferase (IU/L)** | | | | | | | | | |  |  |
|  | | | 1.0 ~ 11.0 | | 49.9 | | | | |  |  |
|  | | | 11.0 ~ 21.0 | | 47.3 | | | | |  |  |
|  | | | 21.0 ~ 28.0 | | 49.2 | | | | |  |  |
|  | | | 28.0 ~ 41.0 | | 51.3 | | | | |  |  |
|  | | | 41.0 ~ 5379.0 | | 52.4 | | | | |  |  |
| **Aspartate aminotransferase (IU/L)** | | | | | | | | | |  |  |
|  | | | | 2.0 ~ 20.0 | 50.6 | | | | |  |  |
|  | | | | 20.0 ~ 24.0 | 49.6 | | | | |  |  |
|  | | | | 24.0 ~ 26.0 | 49.8 | | | | |  |  |
|  | | | | 26.0 ~ 34.0 | 49.7 | | | | |  |  |
|  | 34.0 ~ 3451.0 | | | | 50.3 | | | | |  |  |
| **Preoperative ASA^b^** | | | | | | | | | |  |  |
|  | 1 ~ 2 | | | | | 46.2 | | | | |  |
|  | 2 ~ 6 | | | | 53.8 | | | | |  |  |
| **Preoperative hypertension** | | | | | | | | | |  |  |
|  | No | | | | | 50.7 | | | | |  |
|  | Yes | | | | 49.3 | | | | |  |  |
| **Preoperative diabetes** | | | | | | | | | |  |  |
|  | No | | | | | | | | 49.8 | | |
|  | Yes | | | | | | | | 50.3 | | |
| **Preoperative cardiovascular accident** | | | | | | | | | |  |  |
|  | | | | No | 47.4 | | | | |  |  |
|  | | | | Yes | 52.5 | | | | |  |  |
| **Preoperative asthma** | | | | | | | | | |  |  |
|  | No | | | | | 50.0 | | | | |  |
|  | Yes | | | | 50.0 | | | | |  |  |
| **Preoperative COPD^c^** | | | | | | | | | |  |  |
|  | No | | | | | | 49.6 | | | | |
|  | Yes | | | | | | 50.4 | | | | |
| **Preoperative liver disease** | | | | | | | | | |  |  |
|  | | | No | | 49.8 | | | | |  |  |
|  | Yes | | | | 50.2 | | | | |  |  |
| **Preoperative kidney disease** | | | | | | | | | |  |  |
|  | No | | | | | | | 49.8 | | | |
|  | Yes | | | | | | | 50.2 | | | |
| **Surgery type^d^** | | | | | | | | | |  |  |
|  | No | | | | | 100.0 | | | | |  |
|  | Yes | | | | 0.100 | | | | |  |  |
| **Preoperative tuberculosis** | | | | | | | | | |  |  |
|  | | No | | | 49.8 | | | | |  |  |
|  | | Yes | | | 50.3 | | | | |  |  |
| **Emergency surgery** | | | | | | | | | |  |  |
|  | No | | | | 47.1 | | | | |  |  |
|  | Yes | | | | 53.0 | | | | |  |  |

^a^BMI, body mass index.

^b^ASA: American Society of Anesthesiologists.

^c^COPD: chronic obstructive pulmonary disease.

^d^Surgery type included intrathoracic, intra-abdominal, and supra-inguinal vascular surgery.
